# Exclusive breastfeeding during the COVID-19 pandemic in 17 WHO European Region countries: Ilana Chertok

**DOI:** 10.1093/eurpub/ckac129.190

**Published:** 2022-10-25

**Authors:** I Chertok, R Artzi-Medvedik, M Arendt, E Sacks, M Otelea, C Rodrigues, R Costa, K Linden, M Zaigham, I Mariani

**Affiliations:** 1 College of Health Sciences and Professions, Ohio University, Athens, USA; 2 Ben-Gurion University, Beersheva, Israel; 3 Beruffsverband vun den Laktatiounsberoderinne, Luxemburg, Luxembourg; 4 Johns Hopkins University, Baltimore, USA; 5 Carol Devila University of Medicine and Pharmacy, Bucharest, Romania; 6 University of Porto, Porto, Portugal; 7 EPIUnit, University of Porto, Porto, Portugal; 8 Hei-Lab, Lusofona University, Porto, Portugal; 9 IRT, Porto, Portugal; 10 University of Gothenburg, Gothenburg, Sweden; 11 Skane University Hospital, Lund University, Lund, Sweden; 12 WHO Collaborating Centre, Institute for Maternal and Child Health IRCCS, Trieste, Italy

## Abstract

**Background:**

Maternal experience of labour and delivery is multidimensional and is influenced by a variety of factors.

**Aim:**

to report maternal childbirth experience as described by the women themselves during the COVID-19 pandemic in Sweden using a WHO Standards-based tool adapted for an online survey (Quality of maternal and newborn care-QMNC).

**Methods:**

Women ≥ 18 years of age who gave birth from March 1, 2020 to June 30, 2021 were asked to give voluntary consent to participate in an online survey. The survey included 40 questions on four key domains: provision of care, experience of care, availability of human and physical resources and organisational changes due to COVID-19.

**Results:**

5003 women were included in the analysis. Among those who underwent labour (n = 4528), 46.7% perceived a reduction in QMNC due to the COVID-19 pandemic, 50.7% were not allowed a companion of choice, 62.5% reported that health workers were not always using protective personal equipment and 36.5% rated the number of health workers as “insufficient”. Fundal pressure was applied in 22.2% of instrumental vaginal births and 36.8% received inadequate breastfeeding support. In addition, 18.4% of women did not feel treated with dignity and 6.9% reported some form of abuse. In general, findings were significantly worse among women who did not undergo labour (n = 475).

**Conclusions:**

Swedish mothers’ satisfaction of care provided during childbirth was strongly influenced by many variables. Actions to promote high-quality, evidence-based, patient-centered respectful care for all mothers and newborns are urgently needed.

